# Effect of Glucose Supplementation on Apoptosis in the *Pectoralis major* of Chickens Raised under Thermoneutral or Heat Stress Environment

**DOI:** 10.3390/genes14101922

**Published:** 2023-10-09

**Authors:** Josephine Kwakye, Oluwatomide W. Ariyo, Ahmed F. A. Ghareeb, Evan Hartono, Selorm Sovi, Bikash Aryal, Marie C. Milfort, Alberta L. Fuller, Romdhane Rekaya, Samuel E. Aggrey

**Affiliations:** 1 NutriGenomics Laboratory, Department of Poultry Science, University of Georgia, Athens, GA 30602, USA; josephine.kwakye@uga.edu (J.K.); oluwatomide.ariyo@uga.edu (O.W.A.); ahmed.ghareeb@uga.edu (A.F.A.G.); evan.hartono@uga.edu (E.H.); selorm.sovi@uga.edu (S.S.); bikash.aryal@uga.edu (B.A.); milfort@uga.edu (M.C.M.); alfuller@uga.edu (A.L.F.); 2 Department of Animal and Dairy Science, University of Georgia, Athens, GA 30602, USA; rrekaya@uga.edu

**Keywords:** apoptosis, broiler chickens, heat stress, glucose, mRNA expression levels

## Abstract

Reduced feed intake during heat stress (HS) disrupts glucose homeostasis, thereby resulting in endoplasmic reticulum (ER) stress and triggering apoptosis in chickens. We hypothesize that glucose supplementation could reduce apoptosis in chickens raised under HS. This study comprised 456 28-day-old broiler chickens randomly assigned to four treatment combinations under glucose supplementation and HS. The treatments were TN_0_, TN_6_, HS_0_, and HS_6_ with two glucose levels (0% and 6%) and two temperature levels (25 °C (thermoneutral-TN) and 35 °C (8.00 AM to 8.00 PM, (HS)). After 7 days post-HS, the blood glucose level for the HS_6_ group was higher than for TN_0_, TN_6_, and HS_0_. We studied the mRNA expression of genes and caspase-3 activity in the four experimental groups. The expressions of *GCN2*, *ATF4*, *CHOP*, and *FOXO3a* increased during HS regardless of glucose supplementation, while *PERK* and *MAFbx* increased only under HS with glucose supplementation. We show that under TN conditions, glucose supplementation led to a significant increase in cellular apoptosis in the *Pectoralis* (*P.*) *major*. However, under HS with glucose, the level of apoptosis was similar to that of chickens raised under TN conditions with no glucose supplementation. The utility of glucose to curtail apoptosis under HS should be tested under other intense models of HS.

## 1. Introduction

Heat stress (HS) poses a significant challenge within the poultry industry, especially in regions characterized by high ambient temperatures. The ramifications of HS extend to various physiological and cellular changes in avian species, encompassing disturbances in glucose metabolism, oxidative stress, apoptosis, and the modulation of cellular signaling pathways [1]. Apoptosis, a multifaceted physiological process, holds paramount importance in the preservation of tissue homeostasis, as it undertakes the pivotal role of eliminating compromised or malfunctioning cells, thereby upholding the integral function and structural integrity of tissues at large [2].

Notably, HS has been empirically observed to augment apoptotic occurrences across diverse avian tissue types, including the liver, spleen, and testes [2,3]. Li [4] substantiated this phenomenon by demonstrating that HS in laying hens upregulated the expression of tumor necrosis factor-α (TNFα) and Fas ligand (FasL), thereby instigating apoptosis in the follicles of these layers. Moreover, the prolonged exposure of broilers to chronic HS led to muscular atrophy but the atrophy was attenuated when their dietary intake was supplemented with taurine, resulting in a significant reduction in muscle loss. This effect was attributed to the reversal of apoptosis induced by endoplasmic reticulum (ER) stress and the inhibition of protein breakdown [5].

Heat stress causes reduced feed intake, which leads to deficiencies in both nutrients and energy. This, in turn, triggers the activation of the general control nonderepressible-2 genes (*GCN2*) and adenosine monophosphate-activated protein kinase (*AMPK*) [6]. The *AMPK* gene subsequently activates protein kinase RNA-like ER kinase (*PERK*), which plays a vital role in the cell’s response to ER stress by triggering the unfolded protein response (UPR). Additionally, *GCN2* detects amino acid deficiency and regulates cellular responses, including the inhibition of protein synthesis, promotion of autophagy, and modulation of gene expression, to restore cellular balance [7]. Both *GCN2* and *PERK* phosphorylate elongation transcription factor 2 α (*EIF2α*), triggering apoptosis in birds exposed to HS [8]. Heat stress disrupts glucose homeostasis, leading to alterations in glucose utilization, transport, and signaling pathways in avian tissues due to reduced feed intake [9].

Glucose is a major source of energy for animals, and it has been shown to improve performance, reduce stress, and either enhance antioxidant capacity or accelerate oxidative stress [10,11]. The interconnection between glucose metabolism and apoptosis is complex and multifaceted. Glucose does not only fuel metabolic activities but also serves as a signaling molecule that can modulate various cellular processes, including apoptosis. Glucose supplementation has been shown to defend against apoptosis induced by oxidative stress in hepatocytes [12] and cardiomyocytes [13]. Also, glucose has been shown to reduce apoptosis in the skeletal muscle of mice subjected to hindlimb unloading [14]. Glucose increases antioxidant capacity and reduces oxidative stress in the fatty livers of geese [11]. However, high glucose concentrations have been shown to induce apoptosis in Schwann cells [15] and cardiac microvascular endothelial cells [16].

Despite the growing interest in the effects of glucose on apoptosis, the specific factors that modulate the apoptotic response to glucose supplementation in chickens under different thermal conditions remain to be fully elucidated. Understanding the role of glucose in regulating apoptosis under HS conditions is critical for developing effective management strategies to enhance bird welfare and productivity. Hence, this study investigated the effect of glucose supplementation on apoptosis in the *P. major* of chickens raised under HS or TN environment.

## 2. Materials and Methods

### 2.1. Ethical Clearance

The broiler chickens used in the current study were specifically reared at the Poultry Research Center of the University of Georgia, following the guidelines and regulations set by the Institutional Animal Care and Use Committee, University of Georgia.

### 2.2. Experimental Approach and Sample Collection

The present study used 456-day-old Cobb 500 chicks housed in floor pens. These broilers were fed different starter, grower, and finisher diets based on their respective growth phases. The experiment followed a full factorial design, where the birds were randomly assigned to four treatments with six replicates each, with an average starting weight of about 42 g at day 0.

The experimental design of this study included two factors, a heat stress (HS) and thermoneutral (TN) environment, each with two levels of glucose treatment (0% and 6%). Birds in the TN group were maintained at a constant environmental temperature of 25 °C (77 °F), whilst HS birds were kept under a temperature of 35 °C (95 °F) between the hours of 8.00 am and 8.00 pm from day 28 to 35. The birds had access to water and feed ad libitum, following standard animal care practices [17]. In the treatment group, the drinking water of the birds was supplemented with D-glucose at either a 0% or 6% concentration from day 28 to 35.

Birds were then selected at random from each replicate group at the end of the experiment. Birds were euthanized humanely via cervical dislocation and the *P. major* samples were collected and stored at −86 °C.

### 2.3. Determining Blood Glucose Levels

Blood glucose levels were determined using the VetScan i-STAT^®^ 1 handheld blood analyzer, software version OSi15(A-500.3.36-1) manufactured by Abbott Laboratories, Abbott Park, IL, USA. We followed the manufacturer’s instructions carefully to ensure proper calibration and the accurate measurement of blood glucose levels. Blood samples of 75 µL (two drops) were collected from the jugular veins of birds and loaded onto the i-STAT cartridge. The loaded cartridge was inserted into the i-STAT analyzer, and the analysis was initiated. The i-STAT system utilizes an electrochemical detection method to measure glucose levels in the blood sample.

### 2.4. Gene Expression Analysis via RT-qPCR

Total mRNA was extracted from the *P. major* samples using Trizol reagent according to the established protocol of the manufacturer. Subsequently, complementary DNA (cDNA) synthesis was carried out using the total mRNA as a template with a high-capacity reverse transcriptase kit. Real-time quantitative PCR analysis (RT-qPCR) was conducted to measure the relative expression levels of the target genes. The cDNA samples obtained from the previous step were used as templates for the RT-qPCR reaction in triplicate. The reaction mixture, containing specific primers (Appendix A) with 2× SYBER^®^ (Applied Biosystems, Carlsbad, CA, USA), was prepared according to the manufacturer’s instructions. Amplification was carried out under the following cycling conditions: 95 °C for 15 s and 60 °C for 30 s, for 35 cycles. The threshold cycle (CT) values were determined at the end of each cycle. The relative gene expression levels for general control nonderepessible-2 (*GCN2*), protein kinase-like endoplasmic reticulum kinase (*PERK*), activating transcription factor (*ATF4*), C/EBP homologous protein (*CHOP*), eukaryotic translation initiation factor 2a (*EIF2α*), muscle atrophy forkhead box (*MAFbx*), and forkhead box 3a (*FOXO3a*) were then calculated using the 2^−∆∆CT^ method [18] and analyzed statistically to determine any significant differences between TN_0_, TN_6_, HS_0_, and HS_6_ groups.

### 2.5. Colorimetric Caspase-3 Assay

The activity of caspase-3 was measured using a colorimetric caspase-3 assay kit following the manufacturer’s protocol (ab39401 Abcam, Waltham, MA, USA). Caspase-3 is an executioner caspase that results in apoptosis by breaking down proteins. A total of 24,100 mg *P. major* tissue samples were homogenized in 800 µL of chilled lysis buffer at a pH of 7.5. The resultant mixture was centrifuged at 10,000× *g* for 10 min (4 °C). The supernatant was then kept on ice for 10 min and the accurate concentration of protein was determined immediately using the Pierce BCA Protein Assay Kit according to the manufacturer’s guidelines (Thermo Scientific, Waltham, MA, USA, catalog number: 23227). The absorbance was measured at 562 nm using a Spectra Max 5 microplate reader. In each sample (200 µL), a 2× Reaction Buffer (50 µL) that contained 10 mM dithiothreitol was added in duplicate. This was followed by the addition of 4 mM DEVD-p-NA (5 µL) substrate to each duplicated sample with the exception of the negative control wells. Subsequently, the mixture was incubated in the dark at 37 °C for 90 min. The resulting output was then measured at an optical density (OD) of 400 nm using a microplate reader.

### 2.6. Statistical Analysis

The current study investigated the levels of blood glucose, gene expression changes in the apoptotic pathway, and the activities of caspase-3 in the TN_0_, TN_6_, HS_0_, and HS_6_ groups. The data obtained were analyzed independently for the expression of different genes, blood glucose levels, and caspase-3 activity in all treatment groups (TN_0_, TN_6_, HS_0_, and HS_6_) using two-way analysis of variance (ANOVA) [19]. The generalized linear model (GLM) approach was used. The model used was as follows:

$$y_{ijK}=\mu +a_{i}+b_{j}+{\left(ab\right)}_{ij}+e_{ijK}$$

where $y_{ijK}$ is either the blood glucose composition, relative mRNA expression level, or the caspase-3 activity; *μ* is the overall mean of the response; *a_i_* is the effect of the temperature; *b_j_* is the effect of glucose; (*ab*)*_ij_* is the interaction effect between temperature and glucose; and *e_ijk_* is the random error. The means were separated using Tukey-HSD [19] and significance among treatments was declared at *p* < 0.05.

## 3. Results

### 3.1. Blood Glucose Levels

**Figure 1:**
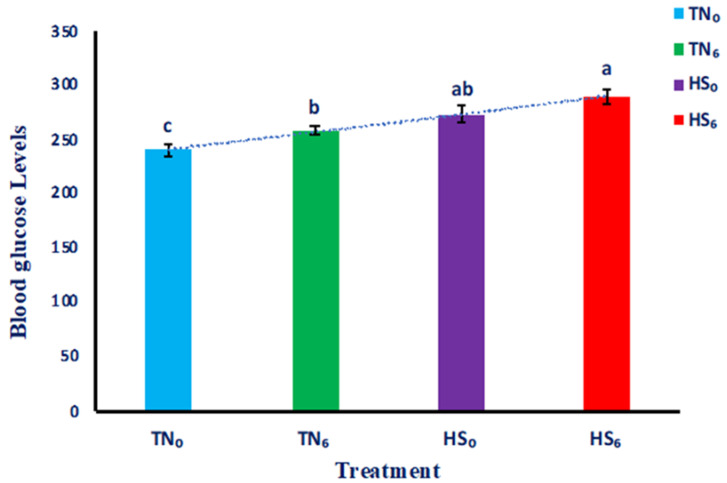
The mean blood glucose levels (±SEM) of birds raised under a thermoneutral or heat stress environment with or without glucose supplementation (*n* = 24). Treatment groups with different connecting letters are considered significant (*p* < 0.05). TN_0_: thermoneutral with no glucose supplementation; TN_6_: thermoneutral with glucose supplementation; HS_0_: heat-stressed birds with no glucose supplementation; HS_6_: heat-stressed birds with glucose supplementation.

The blood glucose levels for TN_0_, TN_6_, HS_0_, and HS_6_ are summarized in Figure 1. The blood glucose levels for the TN groups (TN_0_ and TN_6_) were lower (*p* < 0.05) than those of the HS_6_ group. The HS groups (HS_0_ and HS_6_) had higher (*p* < 0.05) blood glucose levels than the TN_0_ group. Also, the TN_0_ group had the lowest (*p* < 0.05) levels of blood glucose compared with TN_6_, HS_0_, and HS_6_. Additionally, the blood glucose level for TN_6_ was higher (*p* < 0.05) than for TN_0_. However, there were no differences (*p* > 0.05) in blood glucose levels between HS_0_ and HS_6_, or between HS_0_ and TN_6_.

### 3.2. Gene Expression Analysis

**Figure 2:**
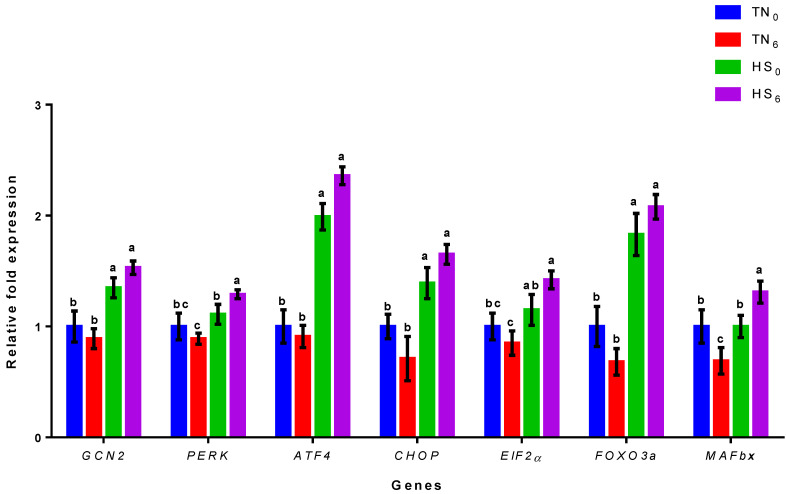
The relative mRNA expressions of genes related to apoptosis in chickens either under a heat stress or thermoneutral environment, regardless of their glucose levels (TN_0_, TN_6_, HS_0_, HS_6_). For each sample (*n* = 18), the RT-qPCR analysis was carried out three times. The output is expressed as 2^−∆∆CT^ (±SEM). Significant groups in the treatment are those with distinct letters (*p* < 0.05). TN_0_: thermoneutral with no glucose supplementation; TN_6_: thermoneutral with glucose supplementation; HS_0_: heat-stressed birds with no glucose supplementation; HS_6_: heat-stressed birds with glucose supplementation.

The relative fold expression levels of *GCN2*, *PERK*, *ATF4*, *CHOP*, *EIF2α*, *FOXO3a*, and *MAFbx* are shown in Figure 2. The mRNA expressions of *GCN2*, *ATF4*, *CHOP*, and *FOXO3a* in the HS groups (HS_0_ and HS_6_) were significantly higher (*p* < 0.05) than those in the TN groups (TN_0_ and TN_6_). However, there were no differences (*p* > 0.05) between the TN_0_ and TN_6_ or the HS_0_ and HS_6_ groups for *GCN2*, *ATF4*, *CHOP*, and *FOXO3a* expression levels. Also, the mRNA expression level of *PERK* and *MAFbx* in the HS_6_ group was significantly higher (*p* < 0.05) than in the TN_0_, TN_6_, and HS_0_ groups. Nevertheless, HS_0_ and TN_6_ were comparable (*p* > 0.05) to the control group (TN_0_) in the expression of *PERK*, but for *MAFbx*, only HS_0_ was similar (*p* > 0.05) to TN_0_. Furthermore, ElF2α showed upward (*p* < 0.05) expressions for HS_6_ when compared with TN_0_ and TN_6_. The mRNA expression of *EIF2α* in the TN_6_ group was significantly lower (*p* < 0.05) than that in the HS groups (HS_0_ and HS_6_) but comparable (*p* > 0.05) to TN_0_.a

### 3.3. Caspase-3 Assay Analysis

**Figure 3:**
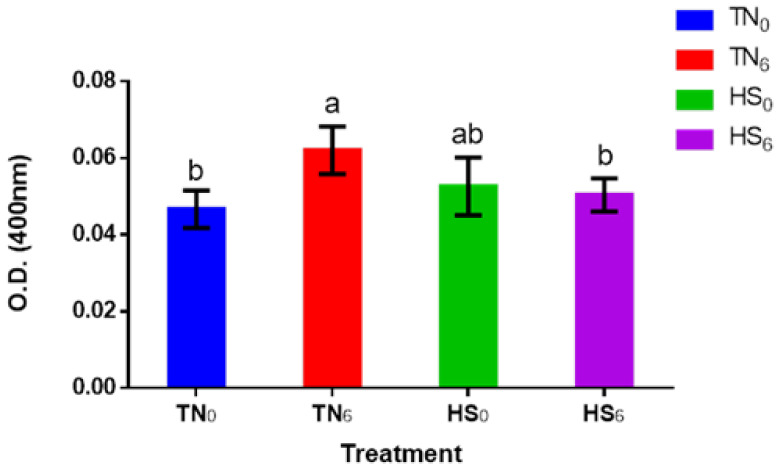
The optical density measurement (±SEM) of caspase-3 activity in the P. major of broilers (*n* = 12) raised under a heat stress or thermoneutral environment, with or without glucose supplementation. The treatment groups with different connecting letters are considered significant (*p* < 0.05). TN_0_: thermoneutral with no glucose supplementation; TN_6_: thermoneutral with glucose supplementation; HS_0_: heat-stressed birds with no glucose supplementation; HS_6_: heat-stressed birds with glucose supplementation.

The caspase-3 activities of TN_0_, TN_6_, HS_0_, and HS_6_ groups are presented in Figure 3. After 7 d post-HS, the OD measured in the TN_6_ group for caspase-3 activity was significantly higher (*p* < 0.05) than in the TN_0_ and HS_6_ groups, but TN_6_ was similar (*p* > 0.05) to that of the HS_0_ group. Also, there was no significant difference (*p* > 0.05) between the HS groups (HS_0_ and HS_6_) and TN_0_ group for the fold increase in caspase-3 activity.

## 4. Discussion

### 4.1. Effect of Exogenous Glucose on Blood Glucose Level

The current study aimed to investigate the effect of HS on blood glucose levels in meat-type chickens, and whether glucose supplementation could modulate blood glucose levels. There was a significant difference in blood glucose levels for the TN_0_, TN_6_, HS_0_, and HS_6_ groups, indicating the impact of HS and glucose supplementation on glucose homeostasis in broiler chickens. Consistent with previous research [20], HS birds exhibited elevated blood glucose levels compared to birds under TN conditions. Heat stress triggers corticosterone that can elevate blood glucose levels by stimulating gluconeogenesis and glycogenolysis in the liver [20].

Broilers receiving glucose supplementation displayed higher blood glucose levels compared to those without supplementation, regardless of their environmental conditions. This suggests that glucose supplementation directly influenced blood glucose concentrations and further contributed to glucose metabolism in broiler chickens. Glucose is the primary fuel source for cellular respiration and serves as a substrate for ATP production through glycolysis and the tricarboxylic acid (TCA) cycle [21]. In the current study, the observed increase in blood glucose levels with glucose supplementation could be attributed to the availability of an exogenous source of glucose, which bypasses the endogenous glucose production pathways in both TN and HS birds. Exogenous glucose is readily absorbed and enters the bloodstream, leading to increased blood glucose concentrations [22,23].

Furthermore, glucose is transported into cells via glucose transporters, which are regulated by insulin signaling. Once inside the cell, glucose can undergo glycolysis to produce ATP or be converted into glycogen for storage in the liver and muscles [24]. Elevated blood glucose concentrations influence hormonal regulation, particularly insulin secretion. Insulin is a key hormone involved in glucose metabolism, as it promotes glucose uptake by cells and inhibits glucose production in the liver [25]. In general, elevated blood glucose concentrations contribute to glucose metabolism in birds by providing a readily available energy source and supporting anabolic processes, as observed in the HS_6_ group of the current study.

### 4.2. Effect of Ambient Temperature and Glucose Supplementation on Apoptosis

The gene expression analysis provided valuable insights into the variations in the mRNA expression levels of *GCN2*, *PERK*, *ATF4*, *CHOP*, *EIF2α*, *FOXO3a*, and *MAFbx* in the HS and TN birds with or without glucose supplementation. The *GCN2* gene is an important regulatory protein involved in cellular stress responses, including apoptosis. The current study revealed the expression of *GCN2* and its downstream effect in relation to apoptosis in birds under different thermal conditions. In the current study, we observed a significant upward expression of *GCN2* in birds subjected to HS regardless of their glucose supplementation levels as compared to the TN groups. The *GCN2* gene is a protein kinase activated in response to cellular stress, particularly amino acid (AA) deprivation. The activation of *GCN2* occurs when there is an imbalance or insufficiency of a specific AA, leading to the accumulation of uncharged transfer RNA (tRNA) molecules [26]. Under normal physiological conditions, when AAs are abundant, tRNAs are charged with their respective AA and translation initiation proceeds. Amino acids are crucial for charging tRNA molecules, which are responsible for delivering AAs to the ribosomes during translation [27]. However, during reduced feed intake, the decreased supply of dietary AAs results in the insufficient charging of tRNA molecules. Consequently, the accumulation of uncharged tRNAs serves as a signal for cellular stress and triggers the activation of *GCN2*, allowing it to phosphorylate its downstream target ElF2α, which initiates apoptosis.

Nevertheless, glucose availability plays a role in modulating the cellular energy status, while energy depletion affects the activation of stress-responsive kinases like *GCN2*. It is worth noting that glucose metabolism can indirectly influence *GCN2* activity [28]. In situations where glucose levels are low or energy reserves are depleted, *GCN2* activity may be upregulated as part of a broader cellular stress response and vice versa [7]. Furthermore, glucose metabolism influences the regulation of AA transporters and AA homeostasis [29,30], potentially affecting *GCN2* activation indirectly through alterations in AA availability. In the current study, *GCN2* expression was not affected by the exogenous supply of glucose, but HS increased *GCN2* expression. The expression of *GCN2* may be more sensitive to nutrients than energy levels as there was no difference between the two HS groups. The mRNA expression of *GCN2* across the treatments suggests that its expression changes was due to HS and not the exogenous glucose supply.

In contrast, *PERK* is a stress sensor activated in response to stress. Heat stress is known to induce cellular stress and trigger the unfolded protein response (UPR), a cellular signaling pathway that aims to restore protein homeostasis in the ER [31]. Under prolonged HS, the UPR fails to restore ER homeostasis, leading to the induction of apoptosis through the activation of *PERK*. Also, immunoglobulin binding protein (BiP), an ER chaperone, normally binds and maintains *PERK* in an inactive state [32]. However, during ER stress, BiP is released from *PERK* due to its binding to misfolded proteins. This dissociation exposes the activation domain of *PERK*. Once released from BiP, *PERK* undergoes autophosphorylation, which involves the phosphorylation of its own residues. This autophosphorylation leads to the activation of *PERK* and its downstream targets, including *EIF2α*, *ATF4*, and *CHOP*, to initiate apoptosis in tissues [32].

In the current study, the *PERK* expression level for HS birds without glucose supplementation (HS_0_) was comparable to that of the control group (TN_0_). However, a study by Seremelis [33] showed an increase in *PERK* expression in HS broiler chickens compared to in TN birds, indicating the activation of UPR. Another study by Ma [34] showed elevated *PERK* expression in HS broilers, also suggesting the induction of the ER stress response. In the current study, *PERK* expression for HS_0_ was slightly higher than for TN_0_ but the difference was not statistically significant. It was expected that glucose would modulate the mRNA expression of *PERK* in HS birds, but that was not the case. Birds under HS conditions supplemented with glucose (HS_6_) showed a significant increase in the expression of *PERK* compared to TN_0_, TN_6_, and HS_0_. It should be pointed out that glucose could act as a substrate for glycosylation, a post-translational modification process that can influence protein folding and stability, thereby leading to protein dysfunction [35]. The current study suggests that the exogenous supply of glucose may have contributed to ER stress, which subsequently induced the mRNA expression of *PERK* in HS_6_ birds.

The activation of both *GCN2* and *PERK* kinases phosphorylate *EIF2α* and *ATF4* [36,37]. In the current study, birds under HS with or without glucose supplementation exhibited an increase in *ATF4* mRNA expression compared to their counterparts reared under TN conditions. The phosphorylation of *EIF2α* activates the integrated stress response pathway (ISR), which modulates apoptotic processes by regulating the expression of apoptotic regulators, caspases, and Bcl-2 family members [33]. One of the key downstream targets of *EIF2α* phosphorylation is the transcription factor *ATF4*, which regulates the expression of a variety of genes involved in stress responses, including those associated with apoptosis. The *ATF4* gene promotes apoptosis under persistent stress conditions by suppressing the expression of anti-apoptotic genes and promoting the transcription of pro-apoptotic genes such as *CHOP*, which plays a significant role in inducing apoptosis during the stress response [38]. The mRNA expressions of *ATF4*, *CHOP*, and *EIF2α* were similar between the two HS groups, suggesting that the exogenous supply of glucose during HS had an insignificant effect on their mRNA abundances.

*CHOP* functions together with *FOXO3a* to induce apoptosis under stressful conditions. The activation of *FOXO3a* via dephosphorylation triggers a series of events that enhance the expression of genes responsible for apoptosis and cell cycle arrest [39]. However, high glucose levels can induce the expression of *FOXO3a*, which, in turn, leads to apoptosis [16]. In the current study, the HS group (HS*_0_* and HS*_6_*) showed an increased level of *FOXO3a* as compared to the TN group (TN*_0_* and TN*_6_*), indicating that *FOXO3a* expressions were mainly due to the effect of HS and not the exogenous supply of glucose. *FOXO3a* influences the mRNA expression of *MAFbx* [40] subsequently leading to atrophy. In the current study, the supplementation of glucose had opposing effects in the TN and HS birds. In the TN birds, glucose supplementation led to a decrease in *MAFbx* mRNA expression when compared with the TN*_0_* birds. However, in the HS birds, the glucose-supplemented birds had higher *MAFbx* expression compared with the TN*_0_* and HS*_0_* birds. Thus, HS does not appear to affect the mRNA expression of *MAFbx*. In a study reported by Zuo [41] from a 21-day continuous HS experiment (28–56 days of age), the expression of *MAFbx* in the *P. major* of broilers was not altered; however, there was a significant increase in the mRNA of *MAFbx* in the thigh muscles of HS birds compared to their control counterparts. In another study, Ma [34] reported an increase in *MAFbx* expression in the *P. major* of broiler chickens subjected to continuous HS for 35 days when compared with their TN controls. Changes in mRNA expression during HS may be affected by the experimental model and the tissues investigated. The duration of the current study was 7 days, and the HS model was cyclical and not continuous as in the studies of Zuo [41] and Ma [34].

We further proceeded to measure the activities of caspase-3 in the *P. major* of broilers under both TN and HS conditions, with or without glucose supplementation. Caspase-3 is a protease enzyme that plays a crucial role in the execution phase of apoptosis [42]. In the caspase-3 assay, the DEVD-pNA sequence was used as a substrate to mimic the specific cleavage site recognized by caspase-3 [42]. Intracellular caspase-3 could be activated by stress signaling molecules, and upon activation, cleaves the peptide bond between aspartic acid (D) and glutamic acid (E) residues. This cleavage event releases the pNA moiety. p-nitroanilide usually absorbs light at a specific wavelength (405 nm). Caspase-3 activity is directly proportional to the level of substrate to be cleaved. Under TN conditions, the exogenous supply of glucose led to an increase in blood glucose level. There was a significant increase in caspase-3 activity in the *P. major* of chickens supplied with exogenous glucose. There are reports that show that apoptosis is among the many cellular responses to high glucose levels [15,16]. High glucose levels cause oxidative and nitrosative stress, leading to apoptosis and necrosis in various cell types [43]. High glucose levels lead to the production of more ATP in the mitochondria. Increased ATP production triggers pro-apoptotic proteins such as cytochrome c to be released from the intramembrane space of the mitochondria into the cytosol [44]. Cytochrome c then facilitates the allosteric activation of apoptosis protease-activating factor 1 (Apaf-1), which forms an apoptosome complex [45]. Each apoptosome has the capacity to assemble apoptotic proteases, subsequently resulting in the catalytic development of caspase-3 and other caspases [46]. Ariyo [47] showed that under TN conditions, supplementary glucose improves feed efficiency by significantly reducing feed intake and marginally improving growth. Despite the improvement in performance, it also leads to a significant increase in apoptosis.

There was a marginal increase in caspase-3 activity in the HS_0_ birds compared with the TN_0_ birds. The mRNA changes in the *ATF4*, *CHOP*, and *FOXO3a* genes in the HS_0_ birds compared with the TN_0_ birds did not translate into significant changes in cellular apoptotic levels. This may be due to the cyclical model and duration of HS. Interestingly, under HS, the supplementation of glucose maintained the apoptosis level, which was similar to birds in the TN_0_ group. The HS_6_ birds had reduced glucose and energy from their feed. Supplementary glucose putatively provided some of the extracellular glucose and energy required to mitigate some of the negative physiological effects of HS and reduced losses in protein biosynthesis. The effect of glucose supplementation on apoptosis should be studied further under a continuous HS model to ascertain its utility in the production system.

## 5. Conclusions

The current study showed that glucose could serve as an extra source of energy for broiler chickens reared under HS conditions. The extra glucose serves as extra energy that can be used to mitigate some of the negative effects of HS. Heat stress with or without glucose supplementation led to an increase in the mRNA expressions of the *GCN2*, *ATF4*, *CHOP*, *EIF2α*, and *FOXO3a* genes compared with birds reared under TN conditions without glucose supplementation. The mRNA expressions of *PERK* and *MAFbx* increased only in the HS group that was supplemented with glucose when compared with the TN non-glucose-supplemented group. The mRNA expression in the selected apoptotic genes in the current study did not translate to apoptosis according to the caspase-3 cellular assay. Under TN conditions, supplementation with glucose led to a significant increase in apoptosis. However, under HS conditions, supplementation with glucose did not change the level of apoptosis when compared with the TN non-glucose-supplemented group.

## Data Availability

The data were obtained during the study and are not available to the public at this time.

## Appendix A

**Table A1:** Primers for Gene Expression Analysis.

Gene	Accession Number	Forward Primer (5′–3′)	Reverse Primer (5′–3′)
*PERK*	XM_420868.6	AGG ATT CTG GCT GTG GTA ATG	CCT TGG TGG AGA AAC AGA TAG G
*GCN2*	XM_004941720.3	GAA TAT CCA GTC GCT GTT CCT AAT A	GGA GTC CAC CTT TCC TTA TCT TC
*EIF2α*	NM_001031323.2	GCC TCC GAT TCC ACC TAT TT	GCC AGT GTA GTG CCA TAT CTT
*ATF4*	NM_204880.2	TTG ATG CCC TGT TAG GTA TGG	CCT GGG TGG TAG GGT TAA ATA G
*CHOP*	XM_015273173.2	TGC TTA GCA GAA TGG GAT GG	GCC ACG CTG ACA CAT GTA ATA
*FOXO3a*	XM_001234495.7	AGT GCA GAA TGA GGG AAC AGG GAA	TGA GAT CCA GGG CTG TCA CCA TTT
*MAFbx*	NM_001039309	GAC AAT GAA CTC AGG GAC ATT TAA C	CGC CAC CTC TAC TGC TTT ATT
*B-actin*	NM_205518.2	AGA CAT CAG GGT GTG ATG GTT GGT	TCC CAG TTG GTG ACA ATA CCG TGT
